# MatureP: prediction of secreted proteins with exclusive information from their mature regions

**DOI:** 10.1038/s41598-017-03557-4

**Published:** 2017-06-12

**Authors:** Georgia Orfanoudaki, Maria Markaki, Katerina Chatzi, Ioannis Tsamardinos, Anastassios Economou

**Affiliations:** 1 Institute of Molecular Biology and Biotechnology-FORTH and Department of Biology-University of Crete, PO Box 1385, Heraklion, Crete Greece; 2 KU Leuven, Department of Microbiology and Immunology, Rega Institute for Medical Research, Laboratory of Molecular Bacteriology, B-3000 Leuven, Belgium; 3 Computer Science Department, University of Crete, Heraklion, Greece; 4 Gnosis Data Analysis PC, Heraklion, Greece

## Abstract

More than a third of the cellular proteome is non-cytoplasmic. Most secretory proteins use the Sec system for export and are targeted to membranes using signal peptides and mature domains. To specifically analyze bacterial mature domain features, we developed MatureP, a classifier that predicts secretory sequences through features exclusively computed from their mature domains. MatureP was trained using Just Add Data Bio, an automated machine learning tool. Mature domains are predicted efficiently with ~92% success, as measured by the Area Under the Receiver Operating Characteristic Curve (AUC). Predictions were validated using experimental datasets of mutated secretory proteins. The features selected by MatureP reveal prominent differences in amino acid content between secreted and cytoplasmic proteins. Amino-terminal mature domain sequences have enhanced disorder, more hydroxyl and polar residues and less hydrophobics. Cytoplasmic proteins have prominent amino-terminal hydrophobic stretches and charged regions downstream. Presumably, secretory mature domains comprise a distinct protein class. They balance properties that promote the necessary flexibility required for the maintenance of non-folded states during targeting and secretion with the ability of post-secretion folding. These findings provide novel insight in protein trafficking, sorting and folding mechanisms and may benefit protein secretion biotechnology.

## Introduction

More than a third of the proteome of all organisms is exported from the cytoplasm^1^. The Sec pathway, the main universal system in cells, is used by >96% of exported proteins in *E. coli* ^1^. Secretory proteins are exported mainly post-translationally^2^. They carry N-terminal signal peptides that are specifically recognized by the SecA receptor of the translocase, the protein-conducting Sec channel^2–4^. After secretion, signal peptides are cleaved at the *trans* side of the membrane^2, 5^. Released mature domains fold or traffic further^5^.

Signal peptides have low sequence but extensive physicochemical property conservation^6^. These include a positively charged N-terminal (n), a central hydrophobic (h), and a C-terminal hydrophilic (c) region that contains conserved signal peptidase cleavage sites^6^. Signal peptides are recognized by robust predictors such as the pioneering SignalP^7^, that predicts their presence and cleavage sites^7–9^ and others that predict protein sub-cellular locations^10, 11^. They include hidden Markov and/or neural network implementations, SVMs and modular architectures^8–10, 12, 13^. Previous studies have demonstrated that the amino acid composition of the whole sequence and surface-exposed residues can serve as predictors of sub-cellular location in prokaryotes and eukaryotes^12–16^. Additional features tested were groups of amino acids (e.g. hydrophobic, polar) and dipeptide content^13^. The datasets used so far mainly rely on rough sub-cellular categories and automatic annotation of general databases such as SWISSPROT and lack in-depth correlation with underlying physicochemical and structural features.

However, fusion to a signal peptide does not guarantee secretion such that several recombinant proteins do not always become secreted^17–19^. Moreover, chimeric signal peptide and mature domain combinations can also be problematic^17–19^. Mature domains contain additional targeting information independent to that of signal peptides and can maintain translocation-competent, non-folded states^19, 20^. Insertion of positive charges in the early mature domain blocks secretion^17, 21–24^ and their removal can optimize export^25^. Chaperones such as SecB^23, 26–28^, Trigger Factor^29^ and SecA^30, 31^ can specifically recognize mature domains.

To recognize and explore mature domain features we developed MatureP, a bioinformatics tool that exploits properties of mature domains to separate them from cytoplasmic proteins. MatureP employs a classification model trained on a dataset of >3,000 proteins with exhaustively annotated sub-cellular topology (Table S1b and c). Model training was carried out without assuming any prior biological knowledge other than that of the aminoacid sequence and topological class (secretory or cytoplasmic). Each protein was represented as a (mathematical) vector of the values of several descriptive features computed from its aminoacid sequence. The classification model was trained with the Just Add Data Bio v0.57 (JADBio; Gnosis Data Analysis; www.gnosisda.gr), an evolution of the BioSignature Discoverer plug-in^32^. JADBio employs a fully-automated machine learning pipeline for producing a classification model given a training dataset, and an estimate of its predictive performance (mean and confidence interval). It also simultaneously performs multiple feature selection, i.e., identification of as many as possible minimal-size feature sets that collectively (multi-variately) contain all predictive information necessary to produce an optimally-predictive classification model.

The selected feature sets provide insight on the properties of the Sec secretome. MatureP revealed that mature domains have unique sequence features that render them a distinct protein class separable from cytoplasmic polypeptides. Secretory proteins represent a balance between optimal secretion features that require the chain to be maintained flexible and non-folded and conservation of the ability to acquire native structure and function after secretion.

## Results

### Definition of minimal mature domain lengths

We focused on *E. coli*, a main biological model, with a fully annotated secretome and well understood, experimentally amenable secretion mechanisms^1^. To determine minimal lengths of mature domains sufficient for export, we examined biochemical and evolutionary evidence.

Truncation analyses suggested that mature domains of lengths of ~50 residues still retain the necessary information for secretion^20, 22, 28, 33^. Moreover, 30 native secreted proteins in the *E. coli* secretome have mature domains of <70 residues (Table S1b; “mature domain length” column, red). Lipoproteins EcnA and B have the shortest (23 and 27 residues, respectively).

We concluded that, N-terminal preprotein stretches of up to 100 (i.e. mature domains of ~65–80) aminoacyl residues contain minimal secretion information and focused our analysis on these (Figure S1).

### Data Analysis Pipeline

**Figure 1:**
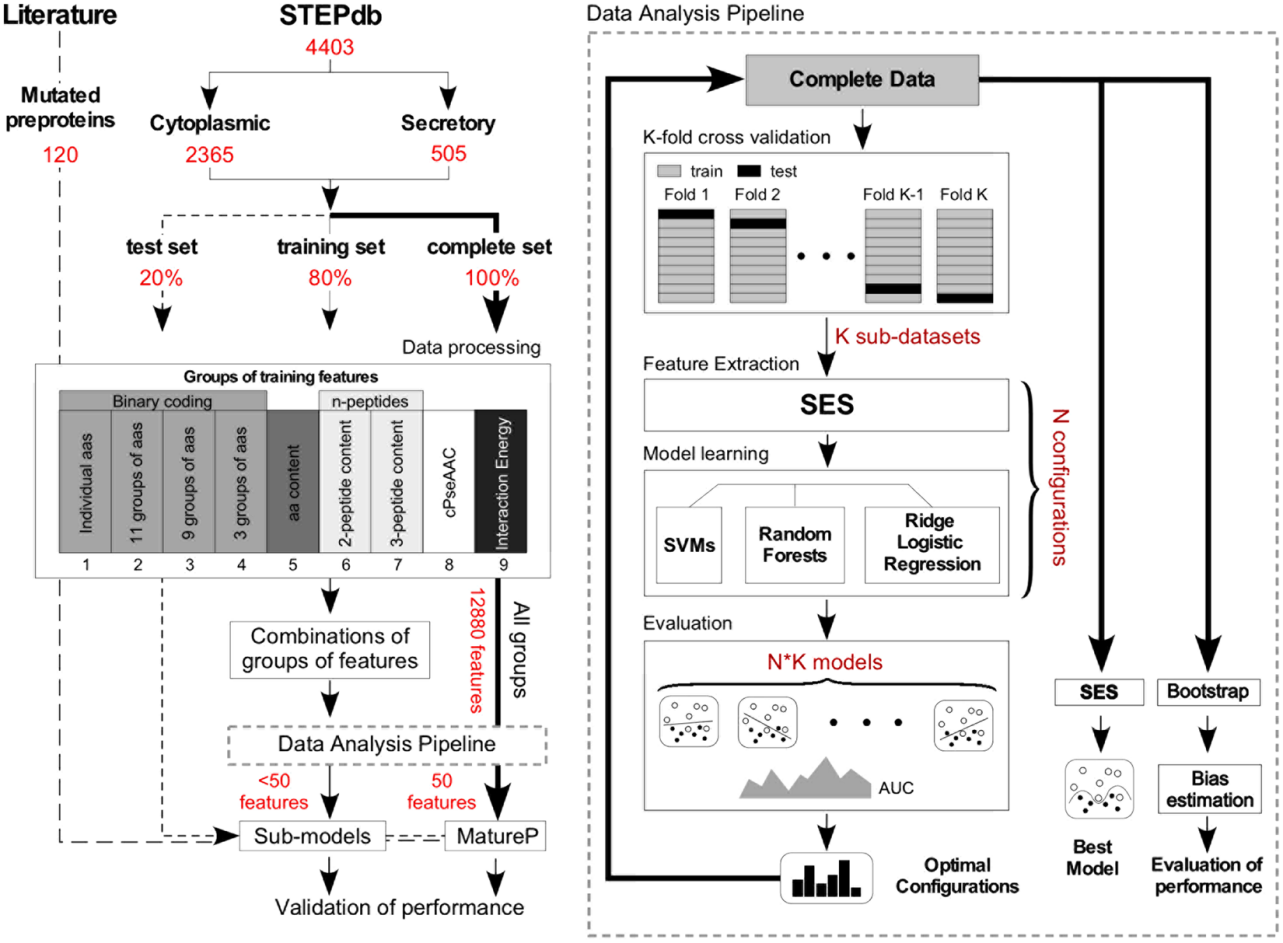
Bioinformatics pipeline of data analysis. Summary workflow of the machine learning process for the separation of secretory from cytoplasmic sequences. First secretory and cytoplasmic proteins from the *E. coli* K-12 proteome were collected based on the subcellular annotation in STEPdb^1^ (Table S1). In total, 2365 cytoplasmic and 505 secretory from eight sub-classes of the cell envelope were defined in STEPdb^1^ (Table S1a). 20% of the dataset (test set) along with 120 mutated preproteins that were collected from the literature, were left outside the training process and were used later on for validation. Raw data (sequences) where first processed and transformed into nine groups of training features (e.g. binary representation of amino acids, cPseAAC). The MatureP model was trained using all data and merging all training features. Data Processing pipeline: The sample set is partitioned to K folds. For each configuration (combination of algorithms and values of their hyper-parameters) and each excluded fold, a model is produced. The average performance of each configuration is then estimated and the optimal one is selected. Subsequently, the final model is trained on all the data using the best configuration. Next, using a boostrapped-based procedure the bias of the performance estimation of the final model is computed; the bias-corrected performance and the final models are returned by the pipeline.

JADBio performs the following functions (Fig. 1; see Methods): (a) (multiple) Feature Selection, (b) Training of classification models, (c) Hyper-parameter tuning of the models, (d) Automated model selection and (e) Unbiased estimation of the mean and the confidence intervals of the final selected model. The pipeline generates several configurations (combinations of feature selection algorithm with classification algorithm for specific values of their hyper-parameters) and subsequently, estimates their performance using stratified K-fold cross-validation. Subsequently, it selects the best configuration and trains with it the final model using all available training data. The last step is to provide an estimate of the performance of the returned model. Because, the model was produced with the best configuration, its cross-validated performance is optimistic (biased); this is a similar occurance to the multiple hypothesis testing. The tool estimates the bias of the performance using a bootstrap method, and removes it to return the final performance estimate and confidence intervals^34^.

The training pipeline was applied to several datasets resulting from representation of the proteins with different sets of features (see below) and the performance was estimated for each (Table S3a,b). Initially, we followed a stratified holdout procedure of splitting data into training and test sets. That is, the same percent of samples (i.e. 20%) for both classes (secretory and cytoplasmic) were held-out and hidden from the tool to serve as an independent test set and to evaluate how accurate is the performance estimation provided by the automated tool. In the majority of these datasets the AUC for the test set was superior than that estimated for the training set (Table S3a,b) suggesting that the data analysis pipeline does not provide optimistic estimations; in fact, it provides slightly conservative performance estimates. This allowed us to train the final model on the complete set of proteins without a hold-out set and to have confidence in the performance estimation provided by the tool.

### Feature visualization and performance analysis

JADBio was used without any restrictions, as well as with the restriction to only search configurations that result in humanly interpretable models, such as linear models and, specifically, Ridge Logistic Regression models. Linear models allow us to investigate, visualise, and better understand the role of the selected features. The unrestricted search, resulting in a possibly non-linear model as optimal, provides an indication of the maximum classification performance that can be achieved on the problem. In contrast, the linear models serve as approximations that allow us to investigate, visualise, and better understand the role of the selected features.

**Figure 2:**
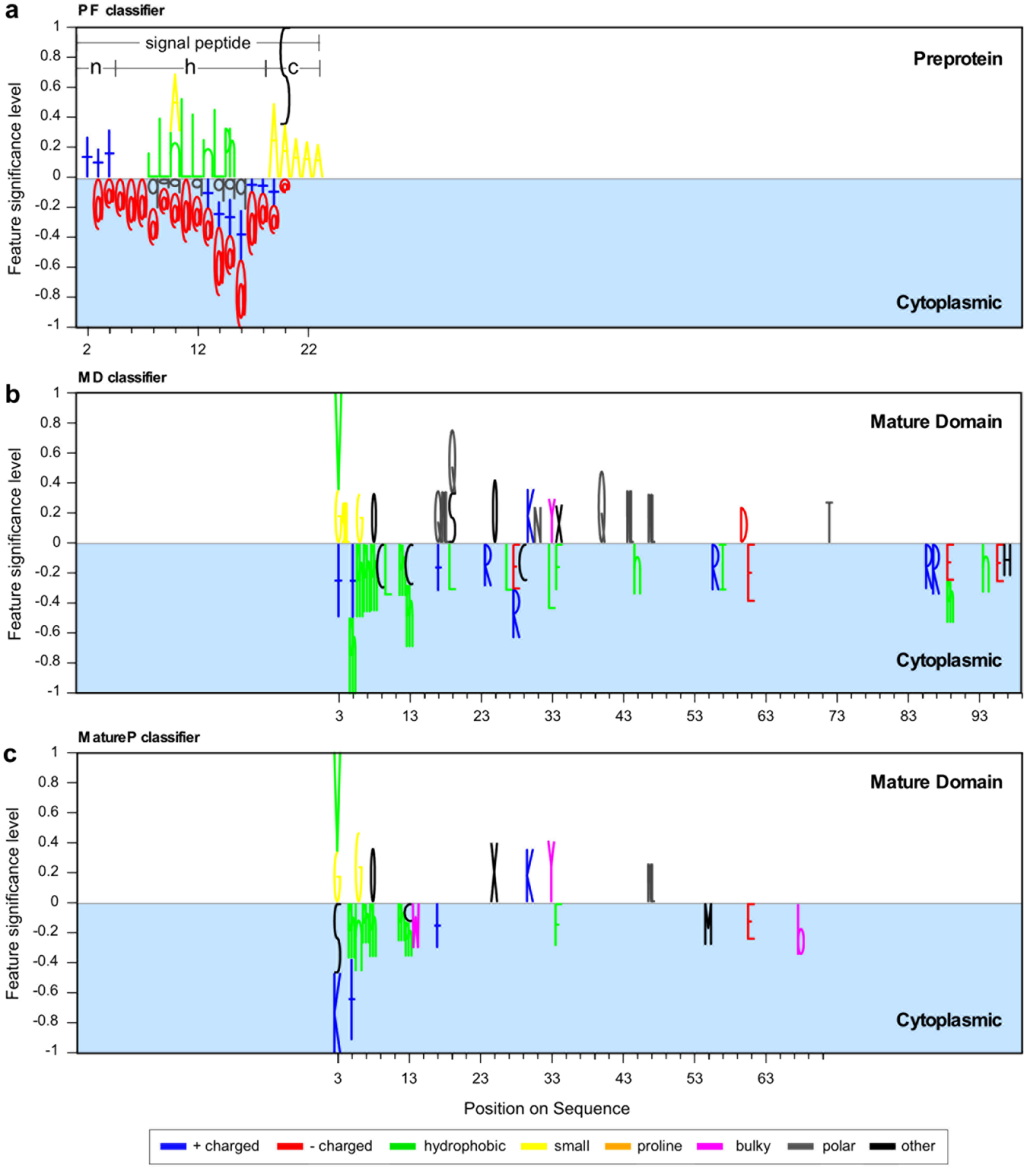
Representation of the selected features. Logo-like representation of the amino acid features taken into acount by each classifier: (**a**) the “preprotein”, (**b**) the “mature domain”, (**c**) and the MatureP. Different features at various positions on the protein sequence can be selected. The features correspond to either individual aminoacyl residues or groups of aminoacyl residues and are represented by a unique letter or symbol (see below). In (**a**) and **(b)** the complete set of the selected features is depicted whereas in (**c**) only the position specific amino acid features are applicable. The coefficients of the two linear classifiers, are the weights of the features which are employed here to represent the classifiers in Logo-like format (see Methods). If more than one features are selected at a position then a stack of symbols is drawn. The height of each stack is indicative of the significance of the position (see Methods). The weights have been normalized from −1 to 1 so that the classifiers are comparable (see Methods). Positively weighted features are selected for secretory whereas negatively for cytoplasmic proteins. In the “preprotein” classifier the most significant features are selected in the signal peptide region. However, there are also features selected in the mature domain region. When the signal peptide is removed (bottom) then more features are selected in the mature domains. A cluster of hydrophobic residues or arginine are disfavoured in the early mature domain (position 1 to 33). Symbols: @: (D,E); + : (K,R); sml: (V,G,A,P); sm: (A,G); h: (I,L,V,M); ph: (L,I,F); b: (Y,W,F); o- (T,S); x:(Y,T,S); pol: (N,Q,C); q: (N,Q,H).

Using the coefficients of the respective linear equations we plotted the most significant features and considered their biological meaning (Fig. 2). For both the non-linear and linear model trainings we tested two different cut-offs (25 or 50) for the maximum number of features used.

JADBio outputs several performance metrics (accuracy, balanced accuracy, sensitivity, specificity, recall, precision). Here we focus our presentation on AUC^35^ (see Methods). The AUC is the metric of choice for measuring performance in binary classification models due to several desirable theoretical properties^35^. An intuitive interpretation of an AUC metric of 90% for example, is that given new proteins, one cytoplasmic (negative) and one secreted (positive), the model has a 90% chance of correctly determining which is which between the two. An optimal threshold on the output of the Ridge Logistic Regression model was applied to achieve equal success for both classes (equal sensitivity and specificity).

### Training features

Nine groups of training features were tested (Fig. 1; Table S3). The first four features are position-specific and correspond to four alternative binary representations: 1. “individual” 2. “relaxed” 3. “compact” 4. “disorder-order” (Table S2, S3). Here the aminoacid sequences are first converted into binary arrays (Table S2). This allows the information of the position and the residue type to be independent from each other (i.e. more than one aminoacyl residues can be selected per position). In the “individual” representation each aminoacyl residue is assigned to a unique 20 bit number. The next three representations were introduced to examine the significance of the physicochemical properties conserved at a specific position of a protein sequence (e.g. charge, hydrophobicity). Given the multi-faceted nature of some residues (e.g. Phe is hydrophobic, aromatic and bulky) we tested alternative organizations of amino acid groupings (Table S2). The next three feature groups aim to reveal potential short motifs. They are a fraction of each type of 1. “residue”, 2. “di-peptide” and 3. “tri-peptide” present in the sequence.

Next we reduced protein sequence complexity using pseudo amino acid composition representation^36^, that preserves part of sequence-order information, and customized it using the hydrophobic character of amino acids (cPseAAC; Supplementary Methods). Finally, we estimated the folding energies of each sequence^37^ (see below).

We trained “preprotein” and “mature domain” classifiers that discriminate secretory preproteins from cytoplasmic sequences and tested the above groups of training features separately or as combinations (Table S3a,b; 28 classifiers). This allowed biological interpretation of the selected features. The final classifier was trained using all features and all data combined (12880; Fig. 1) from which it selects only a small number.

### Detection of signal peptides on preprotein sequences

We first tested our pipeline by analyzing preprotein N-terminal regions including signal peptides and part of early mature domains (~48 to 84 aminoacyl residues). For this we trained classifier #P1 that separates preproteins from cytoplasmic sequences by combining three alternative ways of grouping aminoacyl properties (Table S3b; “individual”, “relaxed”, “compact”). #P1 is a linear classifier with an AUC of 97.1% and is described by only 50 features. This implies that the selected features contribute independently from each other in the classification decision. Therefore, the classification information is rather simple to describe, lies within the primary amino acid sequence and can be summarized by the physicochemical properties of amino acids.

We visualized #P1 features (i.e. continuous stretches), in Logo-like cartoons^38^ (Fig. 2a; Table S3e). The selected individual or grouped residues are drawn per position in the sequence in different colors based on the one letter code notation or unique symbols (for other features; Table S2). The height of the letters/symbols is proportional to their significance in the class prediction. As a measure of the significance of the features we use the rescaled coefficients of the linear equation of the SVM classifier (see Methods). At sequence positions in which more than one feature is selected, we draw a stacked bar with a height equal to the sum of all weights. Features indicated above zero are significantly over-represented in secretory preprotein sequences whereas those below zero (cyan area) are significantly over-represented in cytoplasmic proteins.

The most prominent features that #P1 selects to separate preproteins from cytoplasmic proteins are found within the signal peptide and generally correlate with its features^39^. These results compare well with the top-scoring available methods for the prediction of Sec signal peptides: SignalP^7^, LipoP^8^, Phobius^9^ and the Sec signal peptide-prediction module of PRED-TAT^10^ (see Methods; Table 1). Overall the existing bioinformatics tools outperform #P1 in the preprotein test sets (AUC >99% for all tools). We attribute this difference to the dominance of the conserved signal peptide features being used by all other tools for training optimization.

**Table 1:** Comparison with other bioinformatics tools.

Bioinformatics tool	Performance (%)		
	Train set	Test set¹	Experimental data²
SignalP 4.0^7^	99.61	99.27	51.64
LipoP^8^	99.83	99.71	61.39
Phobius^46^	98.78	98.72	72.08
PRED-TAT^10^	99.66	99.61	62.07
Classifier			
Preprotein (#P1)	97.19	95.51	97.96
MatureP (#M22)	91.46	91.24	85.10
Disorder (#M7)	84.73	84.18	85.86

We measured the performance of four bioinformatics tools: SignalP 4.0 LipoP, Phobius and PRED-TAT on the training, testing and experimental datasets. We used the AUC as a performance metric^35^. AUC depicts relative trade-offs between true positive (benefits) and false positive (costs) and represents the performance of the average classifier (over different classifiers which assume different miss-classification cost ratios).

¹Randomly selected samples (20% of the total sample set; Table S1) which remained unused during the training of the classifiers.

²Experimental data manually collected from the literature (Table S4).

### Features that distinguish mature domains from cytoplasmic proteins

To focus on attributes of mature domains that may be overshadowed in the presence of signal peptides, we trained additional classifiers using only mature domain sequences. To avoid features that are part of known conserved motifs near the signal peptide cleavage site^6, 40^ (Fig. S1d–f), we omitted the first two positions of mature domains, e.g., Cys at position + 1 of all and Glu and Asp at + 2 of some^41^ lipoproteins.

To examine potential conserved motifs on mature domains we trained a “mature domain” classifier that explores the same features as #P1 (Table S3b; #M1). #M1 discriminates mature domains from cytoplasmic proteins with 72.8% AUC using 50 discriminators. By training the equivalent linear classifier of #M1 (Table S3c) we plotted the selected features (Fig. 2b). These suggest that mature domains are enriched in: **a**. polar (Gln, Asn), hydroxyl (Tyr, Thr, Ser) and Asp residues after the first 15 residues. **b**. Glycines at the extreme N-terminus. Mature domains also disfavour several features that are common in the N-terminal regions of cytoplasmic proteins: **a**. short stretches of continuous hydrophobicity (Leu, Iilv, Val, Phe) within the first 15 residues. **b**. positively charged and hydrophobic residues at downstream positions.

### Non-folding propensity in mature domains

To further improve the robustness of our predictors and obtain more structural insight, we next focused on the non-folded states of secretory proteins. It is commonly thought that, like most cytoplasmic proteins, secretory proteins fold rapidly and hence depend on chaperones and their signal peptides to maintain them unfolded^20, 35, 42^. However, experimental data suggest that during targeting to the translocase, mature domains can remain non-folded/disordered in the absence of chaperones or signal peptides^19, 20^ and are predicted to have elevated disorder^5, 43^. M#1 suggested that mature domains and cytoplasmic proteins have different aminoacyl compositions (Fig. 1b; Table S3b,e), implying distinct underlying structural properties. This is also confirmed by M#13 which was trained using solely the amino acid content information (89.85% AUC; Table S3b; Figure S2c). Therefore, we sought to examine whether these differences can be explained in terms of folding energies.

Folding is dictated by a protein’s primary structure^44^ with many inter-residue interactions contributing by lowering the total energy. The total “interaction energy” between the aminoacyl residues of a polypeptide can be estimated by their respective frequencies and an energy predictor matrix P^37^ (see Methods). The P matrix is expressed in energy arbitrary units (au) and defines how the energy of each aminoacyl residue type depends on the presence of other types of residues in the sequence. In other words, P summarizes the inherent property of a protein to fold via the contribution of inter-residue interactions. This approximation has been successfully used for the prediction of intrinsically disordered proteins by IUPred^45^.

**Figure 3:**
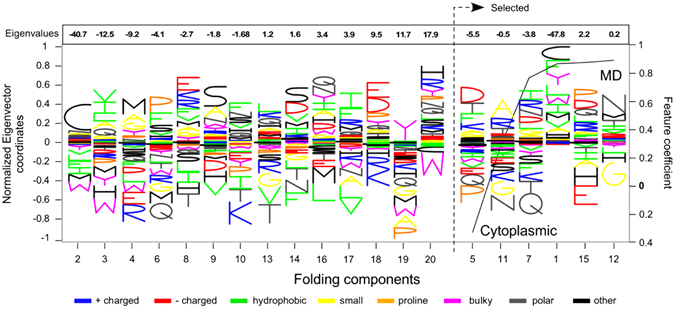
The selected folding features. Decomposition of the energy predictor matrix P to its eigenvectors^37^. Each element ${P}_{i,j}$ of the P matrix, tells how the energy of the residue of type *i* affects the occurrences of the residues of type *j* in the sequence^37^. We name the eigenvectors as folding components (FCs) because they represent a balance between stabilizing and destabilizing aminoacyl residues interactions. We represent each FC as a stack of letters (amino acids one letter code) where the size of each letter is proportional to the corresponding coordinate of the respective eigenvector. The black line denotes the weights of the linear classifier trained using the total energy per folding component (see Methods), termed as the “disorder classifier”. The FCs are rearranged per the weights of the classifier and are numbered in decreasing order of the respective eigenvalues, indicated at the top. Positively weighted FCs are selected for the mature domain sequences. For example, mature domains are composed mostly of Gln, Thr and Lys as the seventh FC dictates.

By decomposing the P matrix to its eigenvectors^37^, it has been proposed that folding can be broken down into its principal components^37^ (Fig. 3). We term the derived eigenvectors “folding components” (or FCs) as each represents a balance between stabilizing (negative energy values) and destabilizing (positive energy values) amino acid interactions (Fig. 3).

We calculated the total interaction energy per FC to segregate the total interaction energy to its components (see Methods) and trained a classifier using only these twenty energy parameters (#M7 classifier; Table S3b). #M7 is linear, requires six out of the twenty FCs and has an estimated AUC for the test set equal to 84.18% (Table S3b). The coefficients are indicative of the type of amino acids that are combined to form a secretory or a cytoplasmic sequence (Fig. 3). For example, FC #1 groups Cys with hydrophobics and aromatics and less of any other type of residue (notice that the eigenvalue is negative and therefore we are looking for positive coordinates). In contrast, FC #8 combines charged amino acids (Arg, Lys and Glu) with hydrophobic and small (Phe, Val, Met and Ala; notice that in this case the eigenvalue is negative).

The most prominent FCs selected for secretory proteins correspond to eigenvectors #12, #15, #1 (Fig. 3; dashed arrow). All three suggest that mature domain sequences are mainly a mixture of residues that ensure higher total interaction energy (i.e. enhanced flexibility). Polar, charged or hydroxyl residues are favoured (FCs #12 and #15). FC #1 follows the same rule; it suggests that mature domains are not a mixture of [Cys, aromatics, hydrophobics] which are stabilizing residues.

The most significant FC for cytoplasmic proteins, #5, is indicative of their different amino acid composition (Fig. 3; right axis, FC selected with a negative coefficient). For this class of proteins there is a residue usage balance such that a negative total interaction energy is achieved (i.e. enhanced stability).

Collectively, these results suggest that mature domains are enriched in destabilizing, while cytoplasmic proteins in stabilizing residues.

### MatureP classifier

The inclusion of additional groups of training features such as cPseACC and folding components yielded significantly improved AUC (~85–90%; Table S3b). By merging all nine groups of features we explored a highly dimensional space (12,880 features) and trained a superior non-linear classifier with AUC 91.5% (Table S3b; #M22). Secretory protein prediction with #M22 compares well with that of the existing tools, which make use of signal peptides (Table 1), particularly in view of the poly-dispersed and weak nature of the features along the mature domain chain.

Because #M22 is non-linear, we produced a corresponding linear model by running the tool with the restriction to return a linear model and examined the role of the selected features. The sign of the coefficients of the corresponding linear model #M22 (Figure S2a) reveals mature domain features that are similar to but significantly expand those of M#1 (only those features that are appropriate for logo-like representation are shown in Fig. 2c): **a**. high content in Asn, Tyr and Ser **b**. high frequency of hydrophobic dipeptides (Figure S2a; cPseACC feature selected “K/D.2”). **c**. Residue usage is dictated by total interaction energy considerations (FCs #15, #7 and #1). Combinations of (Pro, Asp, Gln) or (Thr, Gln, Lys) are favored, implying high chain flexibility (Fig. 3).

We termed the best performing classifier (#M22) MatureP, used it hereafter and developed a new web tool to make it publicly available (current public beta available on-line at www.stepdb.eu/step2/MatureP.php). A combination of #P1 and MatureP classifiers (SP-MatureP) was also developed to run on the online tool, SP-MatureP uses the #P1 classifier for the identification of a signal peptide and estimates its cleavage site. The final decision relies also on the #M22 model which examines the features of the mature sequence. SP-MatureP decides whether a sequence is “cytoplasmic”, a mature or a secretory pre-protein sequence or, more interestingly, if a sequence is a “non-secretory” (i.e. possessing a signal peptide but having a non-compatible mature sequence).

### Validation of MatureP on experimental data

We next sought to validate MatureP against experimental data available in the literature for *in vitro* and/or *in vivo* secretion experiments performed with *E. coli* proteins. To this end we collected a dataset of 120 secretory protein derivatives from 4 secretory proteins that had been mutated in the signal peptide and/or the early mature domain (Table S4). Mutated derivatives were secreted at 0%-105% that of WT proteins (Table S4). Secretion was quantified either by the original authors or by us (see Methods). A very low cutoff for secretion was applied, at least 10% secreted material. MatureP performs very well against these experimental data (85.1% AUC; Table S3a and b) and so does #P1 (97.9% AUC). MatureP was measurably superior to the existing bioinformatics tools (Table 1). This can be attributed to MatureP better exploiting mature domain features that other algorithms ignore^7, 9, 10, 46^. This becomes particularly obvious in derivatives with mutated mature domain regions. The existing tools only detect the signal peptide, they miss-predict the mutated derivatives as being compromised for secretion whereas MatureP does not. Examples of such derivatives are: “1 Pro”, “C5(+4)”, “C6(+6)”, “2AB” of PhoA (Table S4) carrying minor mutations within their mature region which are correctly predicted by #M22 but not by SignalP, Phobius, LipoP or PRED-TAT (the only exception is “2AB” predicted by SignalP). Other derivatives are “Delta23” and “Delta39” of LamB and “BOT” and “D226N” of AmpC (Table S4). Interestingly, the disorder classifier (#M7) has the highest success in predicting these sequences as secretory or not (Table S3b; 93.5%) suggesting that mutated sequences in our dataset may have residues that compromise the propensity of the chains for enhanced disorder.

### Prediction of secretory proteins in other bacterial species

MatureP was trained with the *E. coli* K-12 proteome dataset but protein secretion is a universal, essential process. To test if the features that MatureP selects are universal we measured its effectiveness in predicting secretory proteins from 25 Gram^-^ and 10 Gram^+^ bacteria from various phyla (Table S5a; 7120 and 1361 secretory proteins, respectively). These were identified as being Sec secretory proteins by combining SignalP 4.0^7^, LipoP^8^ and PRED-TAT (see Methods). Only their mature domain sequences were analyzed.

MatureP predicts well secretory proteins from the bacterial cohort test set (Table 2; AUC of 85.7 for Gram^-^; 90% for Gram^+^) suggesting that the aminoacyl residue features selected by this classifier are characteristic of secretory mature domains of the Bacterial domain of life.

**Table 2:** Prediction of Gram^+^ and Gram^-^ secretory proteins from their mature domains.

Classifier	AUC (%)	
	Gram^−^	Gram^+^
Preprotein (#P1)	97.96	97.59
MatureP (#M22)	85.79	90.04
Disorder (#M7)	74.61	82.40^55^

We measured the performance of the main classifiers (“preprotein” and “mature domain”) on a set of secretory proteins predicted in other bacterial proteomes. For this analysis we selected 25 Gram^-^ and 10 Gram^+^ bacteria and collected 7120 and 1361 secretory proteins correspondingly (see Methods; Table S5). The *E. coli* K-12 secretome was excluded since it was used for the initial training.

### Similar Sequences and Data Redundancy

According to Nielsen *et al*.^47^ the training and test sets should be non-redundant and that similar (homologous) sequences should be discarded to avoid overestimating the predictive performance of the classifiers. To further investigate this, we performed redundancy reduction in the original dataset (Table S1b,c) following the procedures used by SignalP^47^ using the algorithm of Hobohm^48^ that performs iterative position specific alignments. This resulted in a non-redundant dataset of 1070 cytoplasmic proteins, 207 preproteins and 247 mature domain sequences (Table S1; see Methods). We also made sure that the wild-type sequences of PhoA, AmpC, LamB and MalE were removed from the training set since their derivatives are included in the experimental evaluation set. New classifiers #P1^NR^ and #M22^NR^ (Table S3b,c) were trained using the non-redundant datasets. The performance was not significantly changed both for the training and test sets (92.76% vs 95.51% for #P1 and 84.80% vs 89.62% for #M22, Table S3a,c) suggesting that our classifiers are unbiased.

Similarly, we sought to examine the possibility that the high AUC values for the Gram^-^ and Gram^+^ bacterial datasets is due to data redundancy. We excluded similar sequences from these datasets (Table S5b,c; see Methods) and recalculated the performance of #P1, and #M22 classifiers (Table S3d). The predictive values were not significantly changed providing corroborating evidence that our training pipeline ensures that the resulting classifiers will be unbiased.

## Discussion

We present MatureP, a classifier that exploits information exclusively from the mature domain region of secretory pre-proteins. MatureP predicts secretory vs cytoplasmic proteins of *E. coli* with an estimated AUC 91.5%. Although MatureP was trained on the *E. coli* proteome it is similarly successful in predicting secretory proteins from mature domain information of 35 diverse bacterial proteomes (AUC of 85–90%). These findings imply that secretory proteins have universal features in the Bacteria that distinguish them from cytoplasmic polypeptides. This finding is remarkable, given that until now these protein groups were considered as having similar structural and folding properties with signal peptide add-ons being the only distinguishing feature of the secreted proteins and being responsible for their maintaining of translocation-competent states^49^. These findings together with recent biochemical and biophysical data that reveal them to have non-structured, expanded states in the absence of any chaperones^19, 50^, suggest that secretory preproteins represent a novel protein class.

No bioinformatics tool currently exists that exploits mature domain sequence information like MatureP does. The closest one is SecretomeP, a neural network-based predictor of non-classical secretors^43^, which are extracellular proteins lacking typical signal peptide sequences and were proposed to be different from the cytoplasmic proteins in amino acid composition, secondary structure and disordered regions. Truncated versions of secreted proteins have also been tested before subtly compromising the prediction accuracy^12, 16^. It was recently proposed that for the Type III secretion system (a protein appendage found in pathogenic bacteria) the respective targeting and recognition signals are distributed along the sequence^51^. MatureP was validated using available experimental data which included mostly derivatives of secretory proteins with either point mutations or extended insertions within the early mature domain region. MatureP is distinctly superior to existing bioinformatics tools in recognizing secretion-compromised, mutated mature domains and not only counting for the presence of a proper signal peptide such as other bioinformatics tools have been trained to do. Model #M22, but in some cases also #P1, (Table 1) can distinguish problematic derivatives (Table S4) from their functional wild-type versions included in the training set (i.e. PhoA, AmpC and LamB). This suggested that our training methods have extracted some form of causality.

Previous studies have found that aminoacyl and dipeptide composition can be used to predict sub-cellular topology^12–16^. However, the preference in certain amino acids has not been attributed to specific physicochemical or structural properties. Here we address for the first time the underlying features being the “cause” rather than the “phenotype” and therefore providing new biological insight. We achieved this by utilizing a more advanced machine learning tool (JADBio) in combination with novel more sophisticated features such as the pairwise interaction energies. JADBio tests three different learning methods: SVMs, Logistic Regression and Random Forests models, selects non-redundant features using Statistical Equivalent Signature^52^ and optimizes several hyper-parameters therefore producing a concise and unbiased classifier.

The comparison of various classifiers trained on different combinations of training features, revealed universal features of the secretory mature domain: polar (Gln, Asn), hydroxyl (Tyr, Thr, Ser) and Asp residues are favored after the first 15 and hydrophobic ones (Leu, Ile, Phe) and Arg significantly disfavored within the first 15 aminoacyl residues. Interestingly, Asp is selected over Glu. Asp (also Asn) has an interesting backbone flexibility profile^53^. It lacks a strong propensity for highly flexible states despite its small size and hydrophilic side chain. This is attributed to the capacity of these residues to form side-chain contacts to backbone hydrogen bonds. Secretory proteins seem to make use of this residue because it can be easily transformed from highly flexible to disordered based on interactions with adjacent amino acids. Some of the particular residues selected are considered as disorder-promoting and may directly reflect on the predicted elevated disorder of mature domains^5^.

The features described above presumably derived from intense evolutionary pressure exerted on the secretome at multiple levels. MatureP reveals the cumulative result of this process. Mature domain sequences have been optimized so as to remain non-folded for the duration of targeting, arrive and dock on a membrane-associated receptor, become secreted through a membrane-embedded narrow channel and still retain all the necessary information on how to acquire their final, catalytically active 3D structure once translocated, or in some cases, additional information for further trafficking^5^. The reverse pressure was exerted on most cytoplasmic proteins leading them to rapid folding routines during or soon after synthesis. Hydrophobic stretches at their N-termini may optimize such folding.

MatureP offers an opportunity to rationally address a long-standing problem in the biotechnology and biopharmaceutical industries. Frequently, recombinant biopharmaceuticals are produced as exported polypeptides fused to signal peptides regarded as “strong”. These “top-performing” signal peptides are chosen based on the abundance of their secreted native mature domains. Nevertheless, signal peptides and mature domains have co-evolved^19^ and hence there exist no “strong” signal peptides outside of their mature domain context. Thus, empirically chosen signal peptides are not always performing best for all their cargo proteins. MatureP provides a rational bioinformatics predictor for these properties. Additional validation of recombinant constructs of industrial interest will help further optimize the algorithm.

## Methods

### Data selection

The 505 Sec-dependent secretory and 2365 cytoplasmic sequences of the *Escherichia coli* K-12 proteome (Table S1) were used during the machine learning analysis. These were collected from STEPdb, a comprehensive database of subcellular classification of *E. coli* polypeptides^1^.

The class of secretory proteins includes eight sub-cellular categories of STEPdb (Table S1A). We included only proteins that utilize the Sec secretion system for their translocation from the cytoplasm to the periplasm^39^ whereas proteins with a Tat signal peptide or the flagellar Type III^5^ were excluded. The cleavage site of the type I signal peptides (e.g. periplasmic proteins excluding lipoproteins) were predicted using SignalP 4.0^7^ and Phobius^46^. The cleavage site of the type II signal peptides (i.e. inner and outer membrane lipoproteins) was predicted with LipoP^8^.

### Description of JADBio Data Analysis Pipeline

JADBio performs the following functions: (a) Feature Selection: feature selection returns multiple statistically-equivalent biosignatures using the Statistical Equivalent Signatures algorithm^54, 55^. Each of the signature consist of a minimal-size set of features (protein characteristics, attributes) that collectively contain all information for optimal classification. Given a signature all other features are either irrelevant (providing no predictive information) or redundant (providing no additional predictive information). JADBio attempts to find as many signatures as possible since the same information may be carried by multiple feature sets. (b) Training of classification models: the tool trains several multivariate advanced and basic machine learning and statistical classification models; namely for classification problems trains Support Vector Machine models (SVMs)^56^ with linear, full polynomial, and Gaussian kernels, Ridge Logistic Regression^57^ models, and Random Forests models^58^. (c) Hyper-parameter tuning of the models: each of the algorithms requires setting the values of several hyper-parameters that tune the sensitivity of the algorithm in identifying patterns in the data and discovering statistical correlations. If an algorithm is too sensitive to pattern finding it may overfit. Alternatively, if it is not sensitive enough it may underfit. In either case resulting in a sub-optimal classification model. Such hyper-parameters are the level of statistical significance in the SES algorithm, the degree of the polynomial kernel in SVMs, the maximum allowable size of the trees in Random Forests and others. Each combination of algorithms and values of the hyper-parameter (called configuration) leads to a different classification model. JADBio automatically tries numerous values performing a grid search in the space of hyper-parameters so as to generate several models and identify the best configuration. (d) Automated model selection: for the tool to select the best final model (corresponding to the optimal configuration) it performs stratified K-fold Cross Validation and estimates the performance of the models resulting from each configuration. It then selects the configuration with the best predictive performance and employs it to train the final model on the complete dataset. Stratified K-fold Cross-Validation works as follows: the dataset is partitioned in K fold (subsets of proteins); stratification implies that the distribution of the class is maintained approximately the same as in the original dataset in each fold. For each configuration a model is trained on all folds but one, and its predictive performance is computed on the hold-out fold serving as a test set. The average performance over all folds provides an estimation of the performance of the average models produced by the configuration. Overall, the tool trains N × K + 1 models, where *N* is the number of configurations to try and K the number of folds (+1 for the final model). In our data analysis N = 35 (combinations of algorithms and values of their hyper-parameters) and K = 10. (e) Unbiased estimation of the mean and the confidence intervals of the final selected model: the performance estimate (even Cross-validated) of the best configuration is optimistic. This is because several configurations are tried and the best is selected^55^. This is a similar and equivalent phenomenon to the multiple-testing problem in hypothesis testing. JADBio uses a bootstrap-based procedure to estimate and remove the bias from the returned performance estimation, as well as produce confidence intervals of performance.

### Ridge Logistic Regression

Ridge Logistic Regression produces an equation of the form Probability(X is a secreted protein) = 1/(1 + e^−z^), where z = *c*_*1*_ *x*_*1*_ + … + *c*_*n*_ *x*_*n*_, *c*_*i*_ the weight (coefficient) of feature *i*, and *x*_*i*_ the value of the *i*th feature of the protein. The decision surface (boundary between the two classes) of this model (where the probability equals 50%) forms geometrically a hyper-plane in *n* dimensions in the feature space (Fig. 1). The higher the absolute value of such a coefficient, the more important is the feature for the final output of the model (assuming feature values are normalized to have standard deviation of 1).

### Receiver Operating Characteristic Curve

The Receiver Operating Characteristic Curve (a terminology historically coming from the development of the radar) graphs the trade-off between the false negative rate (or “one minus specificity”) versus true positive rate (or sensitivity) of classification. Each point on the ROC curves assumes that the classifier uses different misclassification costs ratio or distribution of classes^59^. The AUC is the area below the ROC curve. The AUC represents the average classifier’s cost over different misclassification cost ratios or different distribution of classes^59^.

### Calculating the weights of the selected features

In the logo-like representation of the linear classifiers (Fig. 2) each feature is depicted with a different height based on the magnitude of its importance. The absolute values of the coefficients of the respective linear equations are used as a ranking system. Features selected at the same position appear as a stack of symbols with total height equal to the summation of their respective weights. Negatively weighted features are depicted below the zero axes whereas positive features above. First coefficients are normalized by the standard deviation of the feature. Then the cumulative weight per position is calculated as: ${s}_{i}^{-}=\sum _{k=1}^{n}{w}_{ik},\quad {w}_{ik} < 0$ and ${s}_{i}^{+}=\sum _{k=1}^{p}{w}_{ik},\quad {w}_{ik} > 0$ where ${w}_{ik}$ is the normalized coefficient of the feature *k* at the *i* th position and *n*, *p* the number of negative and positive features selected at the $i$ th position correspondingly. Cumulative weights were normalized from 0 to 1 for positive features and 0 to −1 for negative ones: ${S}_{i}^{-}=\frac{\sum _{k=1}^{n}{w}_{ik}}{\mathop{\max }\limits_{for\,all\,i}|\sum _{k=1}^{n}{w}_{ik}|},\,{w}_{ik} < 0$ and ${S}_{i}^{+}=\frac{\sum _{k=1}^{p}{w}_{ik}}{\mathop{\max }\limits_{for\,all\,i}|\sum _{k=1}^{p}{w}_{ik}|},\,{w}_{ik} > 0$, where ${S}_{i}^{-}$ and ${S}_{i}^{+}$ the normalized sums at the *i* th position. The height of all the features within the same stack is proportional to its weight: ${H}_{ik}=\frac{{w}_{ik}}{\sum _{k=1}^{n}{w}_{ik}}|{S}_{i}|$ where ${H}_{ik}$ the total height of the letter representing the feature *k* at the *i* ^th^ position.

### Estimation of pairwise interaction energy

The total energy of a folded protein depends on: a. the constituent aminoacyl residues and b. the final conformation. As it has been proposed by Dosztányi *et al*. the total interaction energy of a polypeptide sequence can be expressed as a function of its amino acid frequencies^37^. The interaction energy of two types of residues has been approximated by the force fields of solved structures. Then the estimated total energy ${E}_{estimated}$ was expressed as a function of aminoacyl residue frequencies and an energy predictor matrix *P*. *P* was calculated by a system of squared equations of the following form: $\frac{{E}_{estimated}}{L}={\sum }_{ij}^{20}{n}_{i}{P}_{ij}{n}_{j}$ where ${n}_{i}=\frac{{N}_{i}}{L}$ the frequency of the residue type *i*(*L* the polypeptide length). Each element of the *P* matrix, ${P}_{ij}$, tells how the energy of the $i$ th type of residue depends on the occurrences of *j* th type of residue. Decomposition of the *P* matrix to its eigenvectors allows the breakdown of the concept of folding into its principal components (Fig. 3). The total interaction energy per eigenvector was calculated as: ${e}_{k}=sign({\lambda }_{\kappa })\mathop{\,{V}_{k}}\limits^{\longrightarrow}\mathop{n}\limits^{\longrightarrow}$ where *λ*_*k*_ and $\mathop{{V}_{k}}\limits^{\longrightarrow}$ is the *k* th eigenvalue and eigenvector correspondingly and $\mathop{n}\limits^{\longrightarrow}$ is the amino acid composition vector. The total interaction energy values ${e}_{k},\,k=\mathrm{1...20}$ were included in the training procedure (Table S3, interaction energy input features).

### Comparison of MatureP with other tools

We compared the performance of our “core classifiers” with other bioinfomatics tools that predict the existence of signal peptides. SignalP 4.1^7^, LipoP^8^, Phobius^9^ and the Sec signal peptide-prediction module from PRED-TAT^10^ (see Methods). SignalP^7, 60, 61^ consists of two types of neural networks. Phobius and SignalP3.0 are both Hidden Markov Classifiers (HMM)–based tools. They were released to improve miss-classifications of inner membrane proteins as secretory and *vice versa*, sometimes caused by the similarity of the hydrophobic domain of the signal peptide with a *bona fide* transmembrane region. Finally, PRED-TAT^10^ follows an HMM-based modular architecture with sub-models for the prediction of Sec and TAT (Twin-Arginine-Translocation) signal peptides, TM motifs and one for globular proteins.

For SignaP 4.0 we utilized the web-interface and extracted the corresponding Smax values and used them as score values for the calculation of AUC. Smax is the maximum value of S-score along the sequence while S-score is the probability that an amino acid belongs to the signal peptide. Phobius was locally executed using the provided model file. The maximum cleavage site probability of each sequence was extracted and based on these values the final AUC was estimated. LipoP was executed through its web-interface and the probability scores were used for the calculation of the performance. For PRED-TAT that performs combined Sec and Tat signal peptide analyses with a modular HMM-based architecture, we downloaded the Sec and TAT HMM model files and using the provided Perl parser we extracted only the scores that correspond to the Sec HMM module. These scores were used for the estimation of AUC.

### Collection of experimental data from the literature and the validation of the core classifiers

Mutant derivatives of secreted proteins were collected from the literature (see Table S4 for a detailed list). The mutants were classified in three categories: Class I, includes 37 proteins with mutations in the signal peptide. Class II, includes 73 proteins with mutations in their mature domain. Class III, includes 10 proteins with mutations in both the signal peptide and the mature domain. The mutations were insertions, deletions, substitutions or a combination of the two. The experiments were enzymatic and *in vitro* translocation assays in which the secreted material was detected. To be able to compare the differently represented data we established a unified way to quantify secretion. In each reference the data were presented in one of the three following ways: a. the authors had quantified and presented secretion as a percent of the wild type protein. In this case, we used directly the numbers the authors provided. b. the authors had quantified secretion and we transformed it as a percent of that of the wild type. c. the authors presented non-quantified secretion by SDS-PAGE. In these cases we quantified and expressed the data as a percent of that of the wild type protein.

### Secretory proteins in other bacteria

We selected 10 Gram^+^ and 25 Gram^-^ bacteria from various phyla of the bacterial tree (Table S5). The complete proteomes were retrieved from Uniprot^62^ and their Sec secretomes were deduced based on the predictions of three bioinformatics tools: SignalP 4.0^7^ and LipoP^8^ for the prediction of type I (all except liporoteins) and II (lipoproteins) Sec signal peptides correspondingly and PRED-TAT for the distinction between Sec and Tat type signal peptides and also for transmembrane regions^10^. Certain criteria were applied to filter the data. The TAT substrate proteins predicted by PRED-TAT^10^ were discarded irrespective of the predictions of the other tools. From the remaining proteins, we defined as potential lipoproteins those predicted by LipoP to possess type II signal peptides regardless of the prediction results of either SignalP or PRED-TAT. As type I secretory proteins we defined only those that were predicted by all tools: LipoP predicts type I signal peptide instead of type II, PRED-TAT detects Sec type signal peptide and, finally, SignalP also predicts the presence of a signal peptide.

### Redundancy Reduction

The original datasets of *E. coli* K12 (Table S1b,c) and Gram^−^/Gram^+^ bacterial proteins (Table S5b,c) were reduced by removing homologous sequences following the procedures used in by SignalP^47^ and using the algorithm of Hobohm^48^. The blast + suite of NCBI was utilized: makeblastdb command to convert the input fasta files into blast database files and the psiblast command that implements the position-specific iterative basic local alignment search of Altschul *et al*.^63^. Each protein set was searched against itself and cytoplasmic proteins were treated separately from secretory ones both for the *E. coli* datasets and fro those of the other bacteria. We used the percentage of identical matches within the first 100 amino acids as a similarity measure and a cutoff of 40%. Then we sequentially excluded those proteins with the highest number of “neighbours” (algorithm 2 of Hobohm^48^). The remaining proteins are part of the non-redundant sets and are indicated in Table S1b,c for *E. coli* and Table S5b,c, for the other bacterial species.

## Electronic supplementary material

Supplementary info

Table S1

Table S2

Table S3

Table S4

Table S5
