# Supplementary material for: MatureP: prediction of secreted proteins with exclusive information from their mature regions
Source: Sci Rep. 2017 Jun 12;7:3263. doi: 10.1038/s41598-017-03557-4 (PMC5468347; doi:10.1038/s41598-017-03557-4)
Supplement: Supplementary file 1 — Supplementary info [file 41598_2017_3557_MOESM1_ESM.pdf]

SUPPLEMENTARY INFORMATION

MatureP: prediction of secreted proteins with exclusive information from their mature regions

Orfanoudaki, Georgia[1], Markaki, Maria[3], Chatzi, Katerina[2], Tsamardinos, Ioannis[3,4] and Economou, Anastassios[1,2] *

[1] Institute of Molecular Biology and Biotechnology-FORTH and Department of Biology-University of Crete, PO Box 1385, Heraklion, Crete, Greece
[2] KU Leuven, Department of Microbiology and Immunology, Rega Institute for Medical Research, Laboratory of Molecular Bacteriology, B-3000 Leuven, Belgium
[3] Computer Science Department, University of Crete, Heraklion, Greece
[4] Gnosis Data Analysis PC, Heraklion, Greece
*For correspondence: E-mail: tassos.economou@rega.kuleuven.be; Telephone: +32 16 37 92 73

Contents

I. Supplementary Methods

II. Supplementary Figures

Supplementary Figure 1: Entropy logos: Comparison between pre-proteins, mature domains and cytoplasmic proteins – Conserved amino acids at the cleavage site of pre-proteins
Supplementary Figure 2: Linear equivalent classifiers – Selected features

III. Supplementary Tables

Supplementary Table 1: Dataset used in current study (XLS)
Supplementary Table 2 : Binary code representation of amino acids and of their physicochemical properties (XLS)
Supplementary Table 3: Summarizing table of classifiers trained in the current study (XLS)
Supplementary Table 4: Collection of experimental data from the literature for extra validation of our models (XLS)
Supplementary Table 5: Gram$^-$ and Gram$^+$ bacterial species/proteins used for testing the universality of MatureP and preliminary classifiers (XLS)

I. Supplementary Methods

Pseudo amino acid composition

Pseudo amino acid composition format retains some information of the amino acid ordering by calculating 20+λ ranks of sequence-order correlation factors.

Given a polypeptide P that consists of L amino acids P=[R_1 R_2 R_3 ...R_L] }, to avoid completely losing the amino acid ordering information, the pseudo amino acid composition (PseAAC) method was proposed by Chou et al [1, 2]. PseAAC represents a polypeptide by 20 amino acid frequency values and an additional set of τ correlation factors. These correspond to total pairwise products of the physicochemical features (e.g. hydrophobicity) of neighboring amino acids.

Based on Chou et al, PseAAC for a polypeptide P is defined as:

P=[p1, p2,…,p20, p20+1, p20+2,…,p20+λ], λ<L

$$p_u = \begin{cases} \dfrac{f_u}{\sum_{i=1}^{20} f_i + w\sum_{k=1}^{\lambda}\tau_k} & (1 \le u \le 20) \\ \dfrac{w\tau_{u-20}}{\sum_{i=1}^{20} f_i + w\sum_{k=1}^{\lambda}\tau_k} & (21 \le u \le 20+\lambda) \end{cases}$$

where

$$\tau_k = \frac{1}{L-k}\sum_{i=1}^{L-k}\Phi_{i,i+k} \quad (k < L) \quad (2)$$

f_i (i =1, 2,...,20) are the normalized occurrence frequencies of the 20 native amino acids in, τ_j the j-tier sequence-correlation factor computed according to Equation (2) and w the weight factor.

We defined a customized PseAAC (cPseAAC) that was based on the Kyte-Doolittle and Engelman hydrohobicity scales [3, 4] .Therefore, in cPseAAC the multiplied hydrophobicity of two neighboring amino acids is introduced. The distance of neighbours is defined to be λ.

We set w to be 0.5 and λ=60, thus for a sequence of length 100 aminoacyls, 60 τ correlation factors are calculated per hydrophobicity scale.

$\Phi^{KD}_{i,i+j}=H_iH_{i+j}$ H: Kyte Doolitle Hydrophobicity

$\Phi^{En}_{i,i+j}=h_ih_{i+j}$ h: Engelman Hydrophobicity

The first λ parameters that correspond to the normalized summation of pairwise hydrophobicity products based on the Kyte-Doolitlle hydrophobicity scale (KD) are defined as:

$$\tau_1=\frac{1}{L-1}\sum_{i=1}^{L-1}\Phi^{KD}_{i,i+1}$$

$$\tau_2=\frac{1}{L-2}\sum_{i=1}^{L-2}\Phi^{KD}_{i,i+2}$$

.

.

$$\tau_\lambda=\frac{1}{L-2}\sum_{i=1}^{L-2}\Phi^{KD}_{i,i+\lambda}$$

the Engelman (En) based parameters are:

$$\tau_1=\frac{1}{L-1}\sum_{i=1}^{L-1}\Phi^{En}_{i,i+1}$$

$$\tau_2=\frac{1}{L-2}\sum_{i=1}^{L-2}\Phi^{En}_{i,i+2}$$

.

.

$$\tau_\lambda=\frac{1}{L-2}\sum_{i=1}^{L-2}\Phi^{En}_{i,i+\lambda}$$

This representation of the sequences includes the information of the multiplied hydrophobicity of two amino acids that are separated from each other by one and up to sixty

amino acids. In total $2*\min(\lambda, \mathrm{length}-1)+20$ input parameters are introduced in the training process, where length is the size of the amino acids sequence analyzed.

II. Supplemental Figures

Supplementary Figure 1: **Entropy logos: Comparison between pre-proteins, mature domains and cytoplasmic proteins – Conserved amino acids at the cleavage site of pre-proteins**

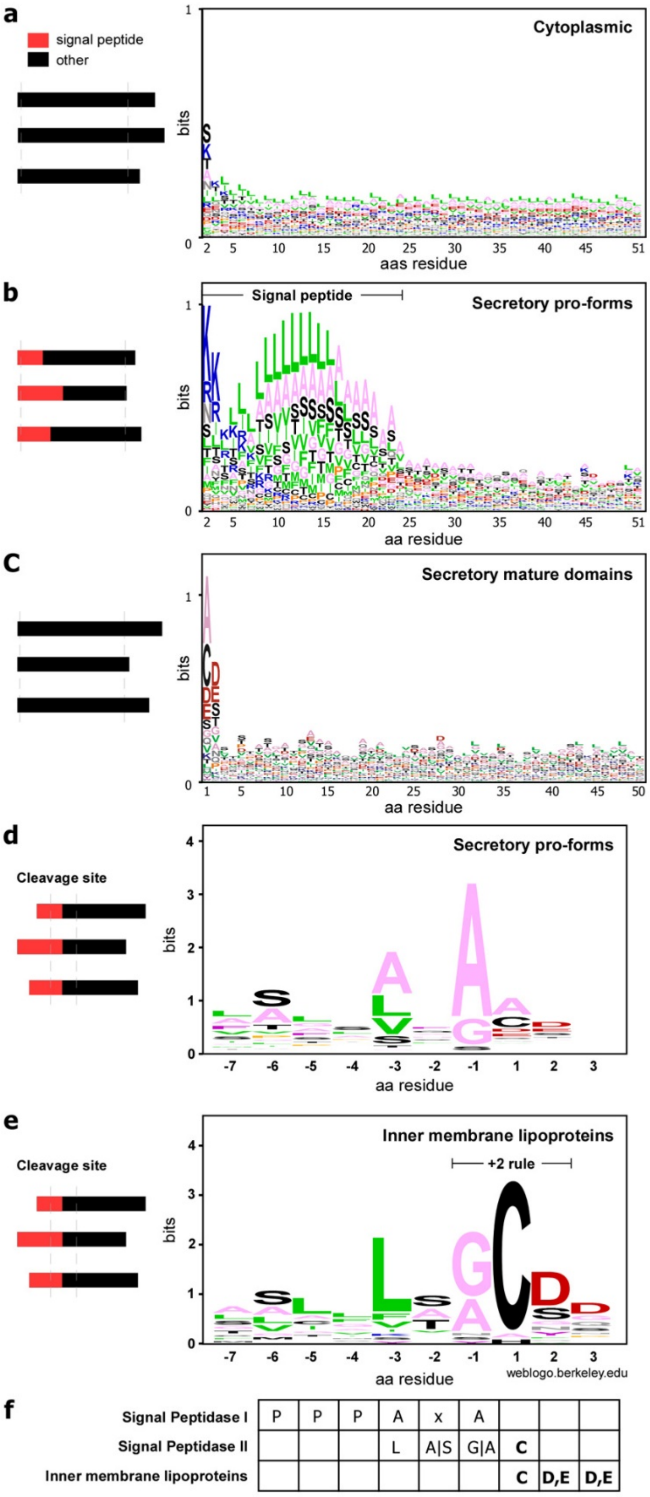

Conserved amino acids per position. Sequences are aligned at either the N-terminal or the signal peptide cleavage site. To the left there are schematic representations of the position of the alignment whereas to the right there are the entropy logos created with WebLogo [5]. **a-c** Cytoplasmic, secretory pro-form and secretory MD proteins. Amino acids after position 50 are omitted for simplicity. The comparison between the three datasets shows a prominent SP motif in the case of the pro-forms. There is a noticeable difference between amino acids preferred in each group of proteins. Cytoplasmic sequences exhibit hydrophobic residues (L,G,A,I) from position +4 and beyond. Negatively charged amino acids (E,D) are selected at positions +1 and +2. **d,e,** Entropy logos of all secretory pro-forms and the subset of inner membrane lipoproteins were

aligned at the cleavage site (see Methods and Supplementary Table 1B). **f** Table summarizing the already characterized motifs on secretory proteins. Periplasmic proteins that are recognized and cleaved by signal peptidase I (SPaseI) follow the -1 and -3 rule, conserved alanines at the respective positions [6-8]. Both inner and outer membrane lipoproteins exhibit 100% cysteines at position +1. Inner membrane lipoproteins tend to have aspartic or glutamic acid at position +2 and +3. These are known to be avoidance signals ("+2 rule") from the LolA pathway that targets lipoproteins to the outer membrane [9, 10].

Supplementary Figure 2: Linear equivalent classifiers – Selected features

Graphical representation of the features selected by the linear equivalent models **a:** #M22, **b:** #M11, **c**. #M13 (Table 3b). #M22 is the final classifier, MarureP, that was trained on all data and using all nine groups of training features (Figure 1, Table S3b). The linear equivalent of #M22 has an estimated AUC 90.51% compared to the 91.46% of the nonlinear one. Classifier #M11 combines six out of the nine groups of training features and has slightly lower AUC that that of the #M22, 89.06% and 90.47% for the linear equivalent and the nonlinear correspondingly. Finally #M13 was trained using only one group of training features, 20 variables representing the amino acid content. This classifier has a remarkable AUC, ~89% for both the nonlinear and the linear versions, even though it uses 14 selected features (Table S3b,c). It suggest that secretory proteins are enriched in hydroxyl residues (T,Y,S) and polar ones (N,Q,D) whereas cytoplasmic in hydrophobics (L,I,F), Cysten and Arginine.

Explanation of acronyms:

X.n: where X is a feature and n the corresponding position on the sequence. A feature can be a single or a group of residues: ***Related symbols:*** @: (D,E); +: (K,R); sml: (V,G,A,P); sm: (A,G); h: (I,L,V,M); ph: (L,I,F); b: (Y,W,F); o: (T,S); x:(Y,T,S); pol: (N,Q,C); q: (N,Q,H)

X%: frequency of the X type residue within sequences (amino acid content)

En.N: Nth correlation factor that is based on Engelman hydrophobicity scale (Supplementary Methods)

K/D.N: Nth correlation factor that is based on Kyte-Doolittle hydrophobicity scale

fc.N: Nth “folding component”. Total interaction energy calculated using the Nth eigenvector of energy predictor matrix P [11]

XY(Z): 2- or 3- peptides

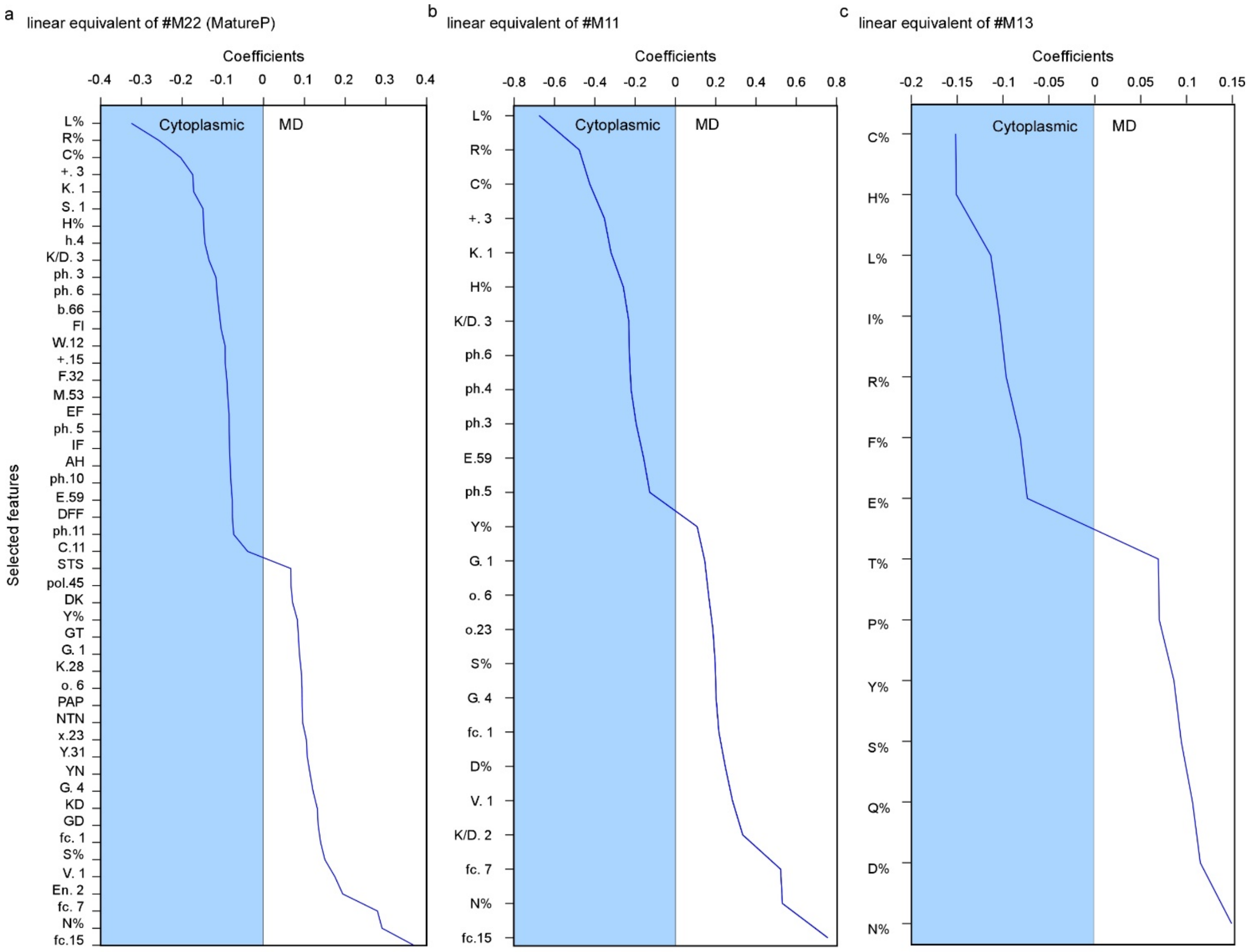

Supplement Figure 2, Orfanoudaki et al

III. Supplementary Tables

Supplementary Table 1: Dataset used in current study (XLS)

a. summary table of the secretory and cytoplasmic proteins used in machine learning analysis. **b.** proteins that use the Sec secretory pathway in order to get completely translocated across the plasma membrane, **c.** cytoplasmic proteins. Based on the annotation of STEPdb [12] they belong to eight subcellular categories and together they add up to 505 secretory proteins **c.** List of 2365 cytoplasmic proteins collected from STEPdb [12]. Similar proteins were discarded after performing redundancy reduction following the procedures defined in the SignalP papers (Nielsen et al., 1997, 1999). The non-redundant datasets were used to test whether the performance of the classifiers is overestimated due to overrepresentation of homologous sequences. Proteins included in the non-redundant datasets are indicated with a separate column.

Supplementary Table 2 : Binary code representation of amino acids and of their physicochemical properties (XLS)

Amino acid sequences were replaced by binary sequences following a binary code representation of individual residues. Alternatively, amino acids were first grouped together on the basis of their physicochemical properties. Since amino acids exhibit multiple properties (e.g. phenylalanine is both hydrophobic and aromatic) three alternative groupings were tested a more “relaxed” (11 groups), one more “compact” (9 groups) and one representing the disorder- and aggregation-prone amino acids. Finally amino acids were organized based on their disorder propensity: ordered (O), medium (M), disordered (D)

Supplementary Table 3: Summarizing table of classifiers trained in the current study (XLS)

Classifiers trained with Just Add Data Bio v0.57 (JAD Bio; Gnosis Data Analysis; www.gnosisda.gr), an automated machine learning tool that combines SES, for feature selection, and SVMs, Random Forests and Ridge Logistic Regression for modeling of the data. The performance is measured as area under the curve percentage (AUC) (depicts relative trade-offs between true positive (benefits) and false positive (costs)).

Three sets of classifiers were trained: **a.** proform ("PF") **b.** mature domain ("MD") and **c.** linear equivalent classifiers (of a and b). **d.** validation of the performance of classifiers using experimental data (Table S5) **e.** selected features (all "PF" and "MD" classifiers)

The "PF" classifiers can predict the secretory preproteins whereas the "MD" classifiers the mature domain sequence of the secretory proteins. In these two cases JAD Bio was used without any restrictions. Then several nonlinear "PF" and "MD" classifier were re-trained with the restriction to only use Ridge Logistic Regression resulting in the linear equivalent models.

Supplementary Table 4: Collection of experimental data from the literature for extra validation of our models (XLS)

Mutant derivatives of secreted proteins were collected from the literature. Secretion efficiencies were quantified from the available published data, either as gel-separated polypeptides or quantified graphs. Mutants could contain a mutation either on the signal peptide, the mature domain or both. This list contains 37 proteins with mutations on the signal peptide (Class I), 73 proteins with mutations in their mature domain (Class II). Class III includes 10 proteins with mutations in both the signal peptide and the mature domain.

Supplementary Table 5: Gram$^-$ and Gram$^+$ bacterial species/proteins used for testing the universlity of MatureP and preliminary classifiers (XLS)

MatureP and all preliminary classifiers (Table S3a,b,c) were trained on curated data from *E.coli* K12 [12]. We put to the test the predictive ability of MatureP in other bacterial species apart from *E.coli* (Table S3d). For this we collected **a.** 25 Gram$^-$ and 10 Gram$^+$ bacterial species. The **b.** Gram$^-$ and **c.** Gram$^+$ secretory and cytoplasmic proteins are listed. The topology of these proteins was predicted SignaP, LipoP, PRED-TAT (see Methods). Similar proteins were discarded after performing redundancy reduction following the procedures defined in the SignalP papers (Nielsen et al., 1997, 1999). The non-redundant datasets were used to test whether the performance of the classifiers is overestimated due to overrepresentation of homologous sequences. Proteins included in the non-redundant datasets are indicated with a separate column.

References

1. Yu, L. et al. SecretP: identifying bacterial secreted proteins by fusing new features into Chou's pseudo-amino acid composition. *J Theor Biol* **267**, 1-6 (2010).
2. Chou, K.C. Using amphiphilic pseudo amino acid composition to predict enzyme subfamily classes. *Bioinformatics* **21**, 10-19 (2005).
3. Kyte, J. & Doolittle, R.F. A simple method for displaying the hydropathic character of a protein. *J Mol Biol* **157**, 105-132 (1982).
4. Engelman, D.M., Steitz, T.A. & Goldman, A. Identifying nonpolar transbilayer helices in amino acid sequences of membrane proteins. *Annu Rev Biophys Biophys Chem* **15**, 321-353 (1986).
5. Crooks, G.E., Hon, G., Chandonia, J.M. & Brenner, S.E. WebLogo: a sequence logo generator. *Genome Res* **14**, 1188-1190 (2004).
6. Perlman, D. & Halvorson, H.O. A putative signal peptidase recognition site and sequence in eukaryotic and prokaryotic signal peptides. *J Mol Biol* **167**, 391-409 (1983).
7. von Heijne, G. Patterns of amino acids near signal-sequence cleavage sites. *Eur J Biochem* **133**, 17-21 (1983).
8. von Heijne, G. Signal sequences. The limits of variation. *J Mol Biol* **184**, 99-105 (1985).
9. Narita, S., Matsuyama, S. & Tokuda, H. Lipoprotein trafficking in Escherichia coli. *Arch Microbiol* **182**, 1-6 (2004).
10. Dalbey, R.E. & Kuhn, A. Protein Traffic in Gram-negative bacteria - how exported and secreted proteins find their way. *FEMS Microbiol Rev* **36**, 1023-1045 (2012).
11. Dosztanyi, Z., Csizmok, V., Tompa, P. & Simon, I. The pairwise energy content estimated from amino acid composition discriminates between folded and intrinsically unstructured proteins. *J Mol Biol* **347**, 827-839 (2005).
12. Orfanoudaki, G. & Economou, A. Proteome-wide subcellular topologies of E. coli polypeptides database (STEPdb). *Mol Cell Proteomics* **13**, 3674-3687 (2014).
